# Clinical Impact of Antifungal Susceptibility, Biofilm Formation and Mannoside Expression of *Candida* Yeasts on the Outcome of Invasive Candidiasis in ICU: An Ancillary Study on the Prospective AmarCAND2 Cohort

**DOI:** 10.3389/fmicb.2018.02907

**Published:** 2018-12-11

**Authors:** Jean-Pierre Gangneux, Muriel Cornet, Sébastien Bailly, Chantal Fradin, Céline Féger, Jean-François Timsit, Olivier Leroy, Boualem Sendid, Marie-Elisabeth Bougnoux

**Affiliations:** ^1^UMR_S 1085 – Inserm, Institut de Recherche en Santé, Environnement et Travail, CHU de Rennes, Université de Rennes, Rennes, France; ^2^CNRS, CHU Grenoble Alpes, TIMC-IMAG, Institute of Engineering, Grenoble INP, Université Grenoble Alpes, Grenoble, France; ^3^Inserm UMR 1137 – IAME Team 5 – Decision Sciences in Infectious Diseases, Control and Care INSERM/Paris Diderot, Sorbonne Paris Cité University, Paris, France; ^4^U995 – LIRIC, Inserm, CHU Lille, University of Lille, Lille, France; ^5^EMIBiotech, Paris, France; ^6^Medical ICU, Paris Diderot University – Bichat University Hospital, Assistance Publique – Hôpitaux de Paris, Paris, France; ^7^Medical ICU, Chatilliez Hospital, Tourcoing, France; ^8^Parasitology and Mycology Unit, Lille University Hospital, Lille, France; ^9^Parasitology-Mycology Unit, Clinical Microbiology Ward, Necker-Enfants-Malades University Hospital, Assistance Publique – Hôpitaux de Paris, Paris Descartes University, Paris, France; ^10^INRA USC 2019, Fungal Biology and Pathogenicity Unit, Institute Pasteur, Paris, France

**Keywords:** invasive candidiasis, *Candida albicans*, *Candida glabrata*, virulence, biofilm, *in vitro* sensitivity, oligomannosides, mannoglycoconjugates

## Abstract

**Background:** The link between *Candida* phenotypical characteristics and invasive candidiasis (IC) prognosis is still partially unknown.

**Methods:** *Candida* strains isolated during the AmarCAND2 study were centrally analyzed for species identification, antifungal susceptibility, biofilm formation, and expression of surface and glycoconjugate mannosides. Correlation between these phenotypical features and patient outcome was sought using a multivariable Cox survival model.

**Results:** *Candida albicans* was predominant (65.4%, *n* = 285), with a mortality rate significantly lower than that in patients with non-*albicans* strains [HR 0.67 (0.46–1.00), *p* = 0.048]. The rate of fluconazole-resistant strains was low (*C. albicans* and *Candida glabrata*: 3.5 and 6.2%, respectively) as well as caspofungin-resistant ones (1 and 3.1%, respectively). Early biofilm formation was less frequent among *C. albicans* (45.4%) than among non-*albicans* (81.2%). While the strains of *C. albicans* showed variable levels of surface mannosides expression, strains isolated from candidemia exhibited a high expression of β-man, which was correlated with an increased mortality (*p* = 0.02).

**Conclusion:** *Candida albicans* IC were associated with lower mortality, and with strains that exhibited less frequently early biofilm formation than non-*albicans* strains. A high expression of β-man was associated with increased IC mortality. Further studies are warranted to confirm this data and to evaluate other virulence factors in yeasts.

## Introduction

Patients hospitalized in intensive care unit (ICU) are at high risk of invasive candidiasis (IC) (Vincent et al., 2009; Kett et al., 2011; Gangneux et al., 2016). Candidemia predominates, associated or not with peritonitis or deep-seated infections (Colombo et al., 2017). The optimal management combines the administration of the right antifungal agent and the control of the infection sources within the appropriate timeframe (Pappas et al., 2008; Cornely et al., 2012). In the large multicenter prospective observational study AmarCAND2, the efficacy of antifungal therapy was evaluated according to the initial drug, its rapidity of introduction, patient-related clinical factors, and the step-down strategy (Bailly et al., 2015; Leroy et al., 2016). The 28-day mortality of IC remained high, up to 40–42%, and was not further jeopardized in case of de-escalation within 5 days.

Little is known on the prognostic value of *Candida* spp. phenotypical features (species, antifungal susceptibility profile, biofilm formation, and glycanic derivatives profile). Yeast mannoglycoconjugates, more specifically through the differential expression of β-1-2 oliganomannosides (β-man) and α-1-2 oligomannosides (α-man), are instrumental for fungal cell wall structure/plasticity, biofilm formation and adhesion (Martinez et al., 1998; Poulain, 2015). The AmarCAND2 study, characterized by a strongly documented clinical database on a large cohort together with centralized mycological analyses on a large number of strains, offered a unique opportunity to describe strains phenotypical characteristics and decipher their prognostic impact.

## Materials and Methods

### Patients and Isolate Collection

This study was ancillary to the AmarCAND2 study on IC in ICU patients (Leroy et al., 2016). We selected patients with proven IC defined as candidemia (≥1 positive blood culture for *Candida* spp.), peritonitis (direct examination or positive culture for *Candida* spp. in a perioperative sample or percutaneous aspiration, excluding drain samples) or deep-seated candidiasis (a positive specimen from a deep organ or usually sterile body fluid). Prognostic factors were evaluated relative to the 28-day mortality.

While the main analysis of AmarCAND2 study relied on mycology results provided by local laboratories, *Candida* spp. strains were also cryopreserved for centralized testings.

### Yeast Identification

Strains were identified using Matrix Assisted Laser Desorption Ionization – Time of Flight (MALDI-TOF) mass spectrometry (Andromas^TM^, Paris, France), according to the manufacturer’s recommendations (see ESM) (Lacroix et al., 2014).

### Antifungal Susceptibility Testing

*In vitro* antifungal susceptibility was performed using Etest^®^ strips (BioMérieux, Marcy-L’Etoile, France) according to manufacturers’ instructions (see ESM) and CLSI M27-S4 clinical breakpoints. Minimum Inhibitory Concentration (MIC) values of echinocandins [caspofungin (CAS), anidulafungin (ANI), and micafungin (MICA)] and triazoles [fluconazole (FLC), and voriconazole (VCZ)] agents were analyzed (see ESM).

### Biofilm Formation

Biofilm formation was assessed using the BioFilm Ring Test^®^ (BioFilm Control^®^, Saint-Beauzire, France) (see ESM) (Olivares et al., 2016). The adhesion strength of each strain was expressed as BioFilm Index (BFI), according to the dedicated software. The software compares the image of the control well to the image of each well and calculates a corresponding BFI value ranging from 0 to 21. Test results defined three groups of strains: no biofilm producers (BFI score within 6 h ≥ 15), low- (BFI ≥ 3 and BFI < 15) or high-biofilm producers (BFI < 3). Values were considered valid when the standard deviation between duplicates did not exceed 10%. Replicates showed a complete categorical accordance within their classification.

### Expression of Surface and Glycoconjugate Mannosides

#### ELISA for the Analysis of Surface Mannoside Expression

The surface mannoside expression was analyzed using enzyme-linked immuno-sorbent assay (ELISA; detailed process in ESM). Briefly, after incubation, the expression of surface β-1,2 and α-1,2 linked oligomannosides (β- and α-Man) was detected using monoclonal antibody (mAb) 5B2, a rat-mouse IgM specific for β-Man, and concanavalin A (HRP-ConA), respectively, (Trinel et al., 1992). A mutant strain of *Candida albicans* expressing no β-Man was used as a control (Courjol et al., 2015). A mannoside score (MS) was defined as the ratio between the optical density obtained for β-Man over that obtained for α-Man. A cut-off MS of ≥3 was arbitrarily determined according to the distribution of α- and β-Man epitopes on whole glycoconjugates.

### Western Blot

Total extracts were obtained from 2 × 10^6^ yeast cells, separated by SDS–PAGE (Leroy et al., 2016)and transferred to nitrocellulose membranes as described previously (Pappas et al., 2009; Bailly et al., 2015). Membranes were then probed with mAb 5B2 followed by alkaline phosphatase conjugated anti-rat IgM (both diluted 1:2000), or HRP-ConA, as previously described (Bailly et al., 2015).

**FIGURE 1:**
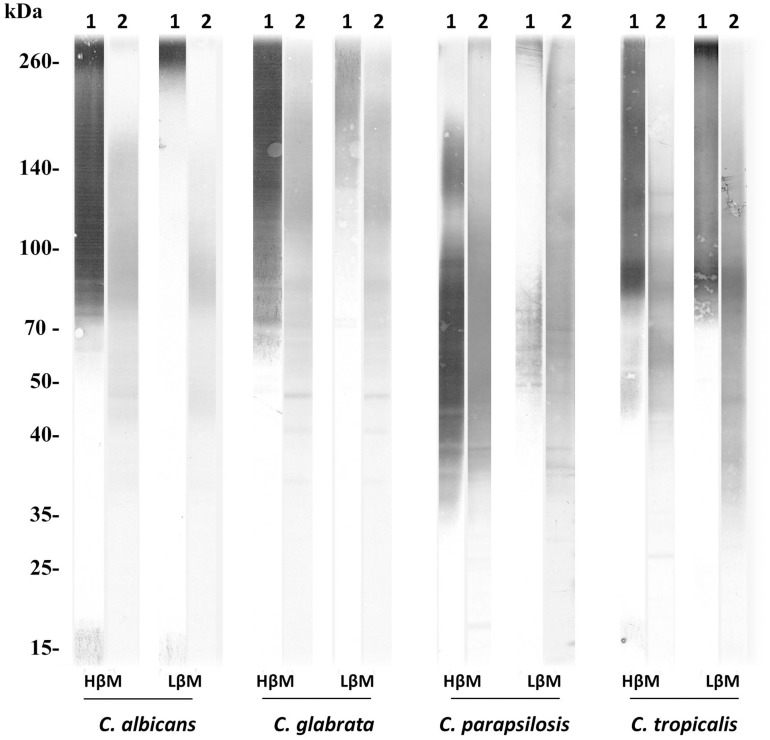
Representative patterns of α- and β-Man expression on glycoconjugates from the four main *Candida* species (HβM, high β-Man profile; LβM, low β-Man profile). Glycoconjugates appear as heterogeneous smears reflecting the glycosylation level of the molecules.

Trinel et al. (1992); Courjol et al. (2015) The solely species considered for glycanic derivatives profile analysis were *C. albicans, Candida glabrata, C. tropicalis*, and *C. parapsilosis*. Expression of α-Man and β-Man on the glycoconjugates was evaluated using the same detection probes (5B2 and ConA). Two patterns were identified on yeast cell glycoconjugates, a high expression (HβM) or a low expression of β-Man (LβM) (representative patterns in Figure 1).

### Statistical Methodology

Data were described using number and percentage for qualitative variables and median and interquartile range for quantitative variables. Comparisons used Chi square test for qualitative variables or Mann-Whitney non-parametric test for quantitative variables. When multiple tests were needed, a Bonferroni correction was applied. Comparisons between methods were performed using the Cohen’s Kappa coefficient with 95% confidence interval. The scale used to assess the degree of agreement was *κ* ≤ 0.2: slight, 0.21–0.40: fair, 0.41–0.60: moderate, 0.61–0.80: substantial, and 0.81–1: almost perfect. Kaplan-Meyer survival curves and Log-rank tests were used to perform univariate survival analysis. Variables associated to mortality (*p*-value threshold of 0.20) were introduced into a multivariable Cox survival model stratified on center. Sub-group analyses were performed according to the species (*C. albicans*) or the infection site (candidemia).

Statistical analyses were performed using SAS v9.4 (SAS Institute Inc., Cary, NC, United States). A *p*-value of < 0.05 was considered as significant.

## Results

**FIGURE 2:**
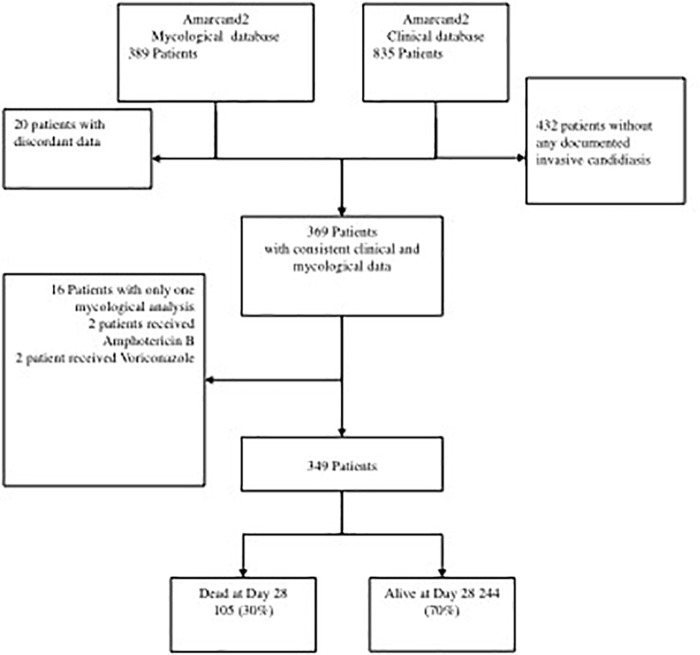
Study flowchart.

Among the patients of the AmarCAND2 study with confirmed IC, 349 were selected for evaluating the correlation between the characteristics of their 436 strains with the 28-day mortality rate and other clinical factors (Figure 2). Overall, 436 strains were isolated from peritoneal fluids (197, 45.2%), blood (159, 36.5%) or other rare sites (80, 18.3%).

### Yeast Species and Site of Infection

The identification provided by each center was well correlated with that of the centralized analysis: Cohen’s Kappa index value of 0.94 [0.9; 0.97]. The MALDI-TOF identification showed the predominance of *C. albicans* (65.4%), followed by *C. glabrata* (14.9%). Other species represented less than 20% [*Candida parapsilosis* (4.6%), *Candida tropicalis* (4.6%), *Candida krusei* (2.5%), and additional species (8%)] (see ESM, Supplementary Table S1). The centralized identification using mass spectrometry on strains of cryptic species allowed the identification of 1 strain of *Candida orthopsilosis*, 1 strain of *Candida bracarensis*, and 4 strains of *Candida dubliniensis*.

*Candida parapsilosis* was more frequent in blood (10.1%) than in peritoneal fluid (1.0%), conversely to *C. tropicalis* and *C. krusei* (peritoneal fluid, 5.6 and 4.6%, and blood, 1.9 and 0.6%, respectively) (see ESM, Supplementary Table S2).

Overall, the 28-day mortality in patients with IC was 32.3% (132/403), and was influenced by the site of infection (Leroy et al., 2016). More specifically, IC due to *C. albicans* were associated with a lower risk of mortality compared to that due to *C. glabrata* and to the other non-*albicans Candida* strains (HR: 0.67 [0.46; 1.00], *p* = 0.048).

### Antifungal Susceptibility

According to the CLSI clinical breakpoints (CBP) or E-Coff values (Maubon et al., 2014), the frequency of resistant strains (R) was rather low (Table 1). Overall, 3.5 and 6.2% of *C. albicans* and of *C. glabrata* were resistant to FLC, and 1 and 3.1% were resistant to CAS, respectively. The MICs results are presented in ESM, Supplementary Table S3.

**Table 1:** Susceptibility results of the main *Candida* species strains of the AmarCAND2 study to the triazoles and echinocandins according to specimen source.

Species^∗^	Categorization [N (%)]														
	
Specimen source (No. of strains)	Fluconazole			Voriconazole			Anidulafungin			Micafungin			Caspofungin		
	
	S or WT	I	R or non-WT	S or WT	I	R or non-WT	S or WT	I	R or non-WT	S or WT	I	R or non-WT	S or WT	I	R or non-WT
*C. albicans*															
Blood	96 (93)	4 (4)	3 (3)	100 (97)	3 (3)	0	101 (98)	2 (2)	0	100 (97)	2 (2)	1 (1)	101 (98)	2 (2)	0
Peritoneal	124 (93)	4 (3)	5 (4)	128 (96)	5 (4)	0	133 (100)	0	0	133 (100)	0	0	132 (99)	1 (1)	0
Other	47 (96)	0	2 (4)	49 (100)			48 (98)		1 (2)	48 (98)	1 (2)		48 (98)		1 (2)
All (285)	267 (94)	8 (3)	10 (4)	277 (97)	8 (3)	0	282 (99)	2 (0.7)	1 (0.3)	281 (99)	3 (1.1)	1 (0.3)	281 (99)	3 (1.1)	1 (0.4)
*C. glabrata*															
Blood	0	22 (96)	1 (4)	22 (96)	0	1(4)	22 (96)	1(4)	0	23 (100)	0	0	5 (22)	18 (78)	0
Peritoneal	0	27 (93)	2 (7)	29 (100)	0	0	29 (100)	0	0	28 (97)	1 (3)	0	7 (24)	21 (72)	1 (3)
Other	0	12 (92)	1 (8)	12 (92)	0	1(8)	13 (100)	0	0	13 (100)	0	0	5 (38)	7 (54)	1 (8)
All (65)	0	61 (93.8)	4 (6.2)	63 (96.9)	–	2(3.1)	64 (98.5)	1 (1.5)	0	64 (98.5)	1 (1.5)	0	17 (26)	46 (70.8)	2 (3.1)
*C. parapsilosis*															
Blood	13 (81)	1 (6)	2(13)	16 (100)	0	0	16 (100)	0	0	16 (100)	0	0	14 (100)	0	0
Peritoneal	2 (100)	0	0	2 (100)	0	0	2 (100)	0	0	2 (100)	0	0	1 (100)	0	0
Other	1 (50)	0	1 (50)	2 (100)	0	0	2 (100)	0	0	2 (100)	0	0	2 (100)	0	0
All (20)	16 (80)	1 (5)	3 (15)	20 (100)	0	0	20 (100)	0	0	20 (100)	0	0	17 (85)	1 (5)	2 (10)
*C. tropicalis*															
Blood	3 (100)	0	0	3 (100)	0	0	3 (100)	0	0	3 (100)	0	0	3 (100)	0	0
Peritoneal	9 (82)	0	2 (18)	11 (100)	0	0	11 (100)	0	0	11 (100)	0	0	11 (100)	0	0
Other	6 (100)	0	0	6 (100)	0	0	6 (100)	0	0	6 (100)	0	0	5 (83)	1 (17)	0
All (20)	18 (90)	0	2 (10)	20 (100)	0	0	20 (100)	0	0	20 (100)	0	0	19 (95)	1 (5)	0


*Susceptibility categories were determined according to the CLSI Clinical Break Points (CBP) as susceptible (S), intermediate (I), and resistant (R) or the E-coff values (ECV) as wild type (WT) and non-wild type (non-WT) when CBPs are not available (Clinical and Laboratory Standards Institute [CLSI], 2012). ^∗^Because of the low numbers of strains within other species than the four detailed in the table, susceptibility data are not detailed.*

The rates of intermediate (I) strains were low, except for *C. glabrata* and FLC (93.8%), and *C. glabrata* and CAS (70.8%). Importantly, among echinocandins, ANI and MICA exhibited much higher susceptible rates (98.5%), underlining the limit of interpretation using CLSI CBP for CAS (see ESM, Supplementary Table S3). Patterns of susceptible (S) and I/R rates for each antifungal agent were similar regardless of the infection site (*p* > 0.05 Chi square test; Table 1).

Based on CLSI breakpoints, the 28-day mortality rate was not different according to the susceptibility profile of the strains involved in the candidemia and peritonitis subgroups (Log-Rank test *p* = 0.16 and *p* = 0.78, respectively).

### Biofilm Formation

**FIGURE 3:**
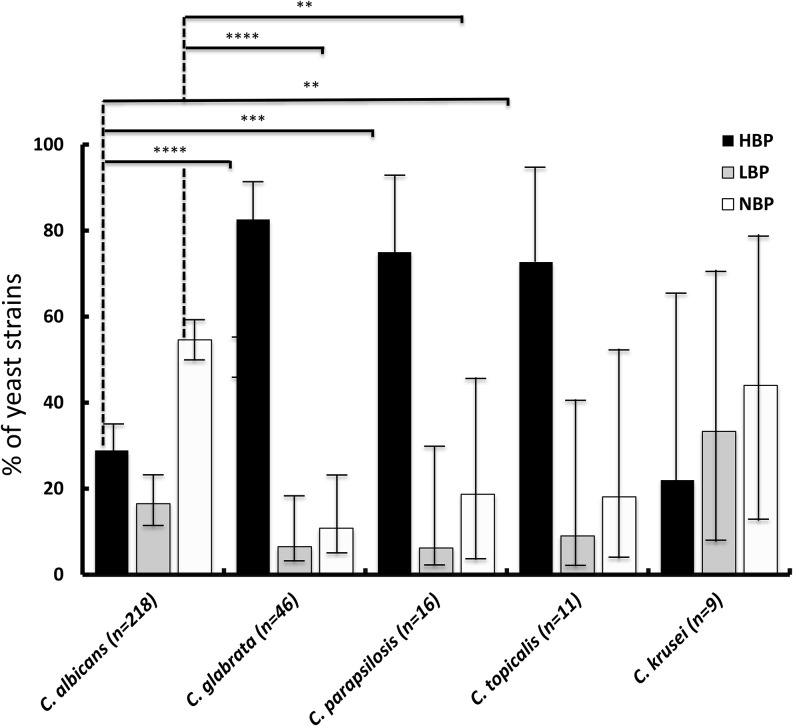
Biofilm production by the study strains of the main species, evaluated based on the biofilm ring test (HBP, high-biofilm producers; LBP, kow-biofilm producers; or NBP, no biofilm producers) [^∗^*p* < 0.008 (significance threshold after Bonferroni correction for multiple tests); ^∗∗^*p* < 0.000; ^∗∗∗^*p* < 0.0000; ^∗∗∗∗^*p* < 0.00000].

Among the 319 strains of *Candida* spp. tested for biofilm formation capacity (Figure 3), 58% (*n* = 181) were biofilm producers. Among these, 27 and 73% were low and high-biofilm producers, respectively, with no statistical difference between strains isolated from blood and from peritoneal sample.

Biofilm production by *C. albicans* was significantly less frequent (99/218, 45.4%) than that by all non-*C. albicans* species (82/101, 81.2%; Chi square *p*-value < 0.01). Furthermore, among producing strains, the proportion of low-producers was lower among non-*albicans Candida* strains (13/82, 15.8%) than among *C. albicans* strains (36/99, 36.4%). Among the main non-*albicans* species, the rate of biofilm-producing strains was higher in strains of C. *glabrata* (89.1%), *C. tropicalis* (81.8%), and *C. parapsilosis* (81.3%) than in *C. krusei* (55.6%) (Figure 3).

There was no statistically significant difference in the 28-day mortality rate according to the biofilm producing ability of the strain among the same species (Chi square *p* = 0.25), whatever the species involved, i.e., *C. albicans* (*p* = 0.25) or C. *glabrata* (*p* = 0.67) (Table 2). In the subset of patients with candidemia, similarly, no link between biofilm production and mortality was identified. No significant association between biofilm production and mortality risk was detected using multivariable Cox regression.

**Table 2:** Comparison of mycological data of the survivors versus the non-survivors at Day 28.

	Survivors at D28 (*N* = 244)	Non-survivors at D28 (*N* = 105)	*P*-value
**Species**			
*C. albicans*	170 (69.7)	71 (67.6)	0.7266
*C. glabrata*	35 (14.3)	14 (13.3)	
*C. parapsilosis*	13 (5.3)	5 (4.8)	
*C. tropicalis*	9 (3.7)	3 (2.9)	
Other species	17 (7)	12 (11.4)	
**Origin of the sample**			
Other	37 (15.2)	8 (7.6)	0.0054
Blood sample	85 (34.8)	55 (52.4)	
Peritoneal sample	122 (50)	42 (40)	
**I/R (CLSI)**			
24 h	38 (15.6)	12 (11.4)	0.3107
**Biofilm data**			
Ratio dose/CMI	41.6 [18.9; 72.3]	48.3 [21.3; 70.3]	0.5696
Biofilm: High	100 (41.0)	34 (32.4)	0.3518
Biofilm: Low	49 (20.1)	21 (20)	
Biofilm: No	95 (38.9)	50 (47.6)	
Biofilm Score (Continuous)	7.3 [0; 19.9]	13.1 [0; 20]	0.1195
**Mannoside data**			
Ratio > 3 and HβM	115 (47.1)	60 (57.1)	0.3697
Ratio > 3 and LβM	30 (12.3)	11 (11.4)	
Ratio ≤ 3 and HβM	56 (23.0)	19 (18.1)	
Ratio ≤ 3 and LβM	43 (17.6)	14 (13.3)	
Ratio ≤ 3	99 (40.6)	33 (31.4)	0.1061
Ratio > 3	145 (59.4)	72 (68.6)	
LβM	73 (29.9)	26 (24.8)	0.3271
HβM	171 (70.1)	79 (75.2)	
Ratio ≤ 3 and LβM	43 (27.2)	14 (18.9)	0.1713
Ratio > 3 and HβM	115 (72.8)	60 (81.1)	
Ratio mannoside (Continuous)	5.2 [1.3; 16.3]	6.3 [2; 28.2]	0.0548
Log ratio mannoside (Continuous)	1.7 [0.3; 2.8]	1.8 [0.7; 3.3]	0.0548
HβM	117 (70.1)	79 (75.2)	


*HβM: high β-man profile; LnβM: low β-man profile.*

### Glycanic Derivatives Profile

A total of 387 yeast strains were analyzed for the expression of a α- and β-Man. Large differences in MS were observed among *Candida* species; the highest values were observed for *C. lusitaniae, C. tropicalis, C. dubliniensis*, and *C. albicans* (see ESM, Supplementary Table S4). There were no significant differences according to the sampled body site. Western blot analysis revealed rates of strains with HβM and LβM profiles at 68.3%/31.7%, 75%/25%, 84.2%/15.8%, and 87.5%/12.5% for *C. albicans* (*n* = 259), *C. glabrata* (*n* = 56), *C. parapsilosis* (*n* = 19), and *C. tropicalis* (*n* = 16), respectively, (*p* = 0.0004), while there was no significant difference between the HβM and LβM profiles of strains according to the sampled body site (*p* = 0.16). Unexpectedly, the analysis of the correlation between MS and antifungal susceptibility showed that yeast phenotypes with MS ≤ 3 were significantly associated with reduced susceptibility (I/R profile) (*p* < 0.001), while the distribution of strains able or not to produce biofilm was significantly different according to MS values (*p* = 0.0054).

**FIGURE 4:**
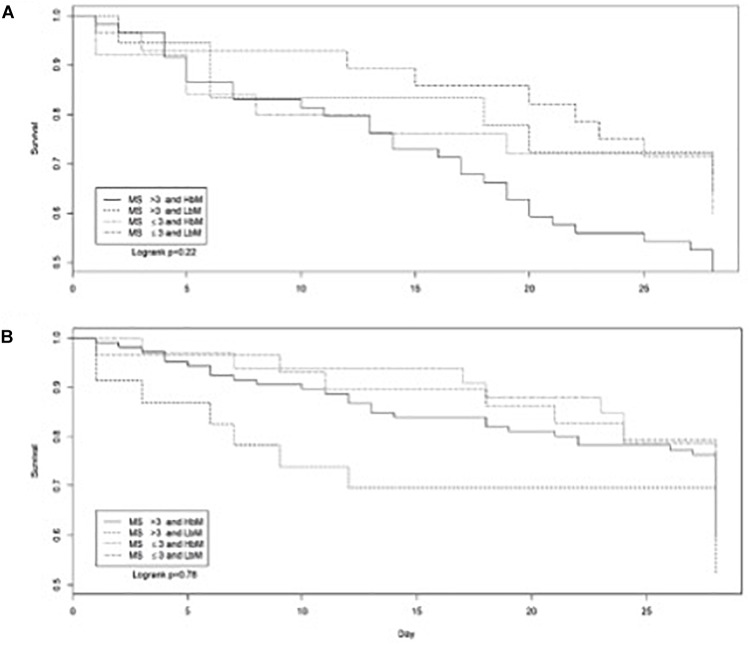
Survival curves for patients with candidemia **(A)** or invasive candidiasis proven by the isolation of *Candida albicans, Candida tropicalis, Candida glabrata, Candida parapsilosis*, and *Candida dubliniensis* from the peritoneum or other tissue or body fluid samples **(B)** according to the MS and β-Man profiles (restricted to the strains characterized regarding HβM and LβM) (1, MS > 3 and HβM; 2, MS > 3 and LβM; 3, MS ≤ 3 and HβM; 4, MS ≤ 3 and LβM).

In patients with candidemia, a high MS combined with a HβM profile was associated with an increased 28-day mortality (Chi square *p* = 0.02) (Figure 4). Of note, most candidemia were due to *C. albicans* (64.8%), which is a species frequently harboring high MS value (75% of *C. albicans*) and HβM profile (68.3% of *C. albicans*). The β-Man profiles and MS were also different in patients initially treated with candins while having an IC due to strains of *C. albicans*; survivors had more often a high MS (Chi square *p* = 0.03).

However, when considering MS as a continuous variable for all yeast species, values tended to be higher in strains isolated from patients who died vs. strains collected from survivors (*p* = 0.12; ESM, Supplementary Table S3).

## Discussion

This study evaluated the phenotypical features of a large collection of clinical strains of *Candida* spp. involved in proven IC in well documented critically ill patients enrolled in the AmarCAND2 cohort study (Leroy et al., 2016).

Our study showed the persisting leading role of *C. albicans* in IC of French ICU patients. In France, the rate of IC caused by *C. glabrata* remained rather low and stable in recent studies, including in the previous AmarCAND1 study (Leroy et al., 2009; Baldesi et al., 2017). This trend is also observed in other Northern European countries (Das et al., 2011; Arendrup et al., 2013) as opposed to Southern Europe (Montagna et al., 2014). Of note, we analyzed only the first isolate that confirmed the diagnosis, whereas IC due to *C. glabrata* are usually more frequently diagnosed in patients with prior exposure to antifungal agents. This restriction to the first isolate may also explain the rather low rates of resistance observed.

Our study did not evidence a higher rate of one specific *Candida* species in one body site compared to another, except for *C. parapsilosis* which was mostly associated with candidemia.

The multivariate logistic regression showed a lower mortality risk of *C. albicans* in the subgroup of patients with candidemia as compared to *C. glabrata* and other non-*albicans Candida* strains, whereas higher rates of crude mortality were reported in patients with candidemia due to non-*albicans Candida* strains from other ICU studies (Bougnoux et al., 2008; Montagna et al., 2014). However, in studies with a clinical set of data sufficiently detailed for conducting multivariable logistic regression, findings are variable, some identifying a higher independent risk of death for candidemia due to non-*albicans* species (Dimopoulos et al., 2008; Lortholary et al., 2014), while the species of the *Candida* strain was not an independent risk factor for another study (Klingspor et al., 2015).

The level of resistance to the five antifungals tested in this study was low in *C. albicans* strains, similarly to the one of the AmarCAND1 study performed 5 years before (Leroy et al., 2009). The rate of FLC resistance was not so low among *C. parapsilosis* (15%) and *C. tropicalis* (10%), however, the number of strains was small. We confirmed the higher MIC levels displayed by the *C. glabrata* strains for CAS than for ANI or MICA (Espinel-Ingroff et al., 2016), leading to major discrepancies in the SIR categorization of this species between the three echinocandins. These discrepancies support the recommendation to avoid using the CLSI CBP for CAS and *C. glabrata*, and more generally to avoid testing CAS for *Candida* spp. with *E*-tests, due to unreliable MICs results (Maubon et al., 2014; Espinel-Ingroff et al., 2016).

The ability to form biofilms by *Candida* strains *in vitro* was variable depending of the species. Strikingly, *C. albicans* was the species the least prone to form biofilm in comparison to other *Candida* species. Furthermore, the biofilm production ability was highly variable among *C. albicans* strains. These results are of interest, as few studies have evaluated the biofilm formation by clinical strains of *Candida* involved in IC. Some studies focused on candidemia (Pongracz et al., 2016; Rajendran et al., 2016). Of note, in these studies, biofilm production was evaluated over a 24-h period, while we evaluated the early production of biofilm. Indeed, compared to techniques based on biomass detection, such as the chromogenic XTT assay and Cristal Violet staining, our technique evaluates the first step of biofilm formation process, i.e., the adhesion step. Detecting this step by using a quick test might bring an instrumental contribution to the decision process regarding the management of indwelling devices such as intravenous catheters.

To the best of our knowledge, our study is the first one to evaluate the early stage of biofilm formation on a large collection of strains involved in IC not limited to bloodstream infections, and specifically in ICU patients. As in studies on biofilm formation by *Candida* strains of bloodstream infection (Pongracz et al., 2016; Rajendran et al., 2016), we showed that the capacity to adhere and to develop to substrate surfaces was more often observed in non-*albicans Candida*. However, unlike one of them (24), we did not identify any link between biofilm production and mortality in the subset of patients with candidemia. This discrepancy can be due to differences in the management of antifungal therapies or intravenous devices removal in the two studies.

Mannosides expression varies according to the strain, growth conditions and cell stage growth (Prill et al., 2005). For the first time, we showed that the level of β-mannosides expression on *Candida* species was first correlated to other yeast phenotypic features such as biofilm formation and susceptibility to antifungals; and second to the infection outcome. Altogether, our findings suggest that β-Man glycoconjugates expression may be instrumental for *Candida* pathogenicity, at least at systemic level. Previous studies have evidenced the implication of β-Mans in *Candida*-host interaction and virulence mechanisms and in host immunomodulation (Jouault et al., 1995). These data emphasize the need for further analysis of the role of mannosides expression in the outcome of patients with IC and of the impact of the expression of mannosides on antifungal susceptibility.

This study had some limitations. First, it was conducted in one country and results may not be fully extended to other countries. Second, it was an observational study with sample collection at the inclusion, without any study-specific intervention. Finally, *in vitro* sensitivity testing was performed using *E*-test strips without confirmation using a standardized CLSI or EUCAST method. However, this represents the way antifungal susceptibility is performed in routine in French hospitals, and results presented here were all confirmed results from centralized experienced laboratories.

Importantly, this study is the largest study to evaluate the correlation between some virulence factors and clinical outcome in a large cohort of ICU patients with IC, and it provided some interesting data. Candidemia due to *C. albicans* was associated with a lower risk of mortality compared to non-*albicans Candida* strains and strains exhibited less frequently early biofilm formation than non-*albicans* strains. *C. glabrata* was not overrepresented in intraperitoneal infections that did not constitute a reservoir of resistant strains. Regarding more original virulence factors investigated here, we interestingly showed that mannosides expression on *Candida* species was correlated to biofilm formation and susceptibility to antifungals, and that the high surface expression of β-Man tended to be associated with a poorer 28-day outcome. Further studies on yeast virulence factors in various patient populations are warranted to more precisely evaluate their prognostic value.

## Author Contributions

All authors listed have made a substantial, direct and intellectual contribution to the work, and approved it for publication.

## Conflict of Interest Statement

JP-G received research and travel grants from Astellas, Gilead, MSD, and Pfizer. MC received research and travel grants from Basilea, Gilead, MSD, and Pfizer. CFe received grants from MSD. J-FT received research and travel grants from MSD, Pfizer, Gilead, Biomerieux, Bayer pharma. OL has given lectures for symposia set up by Abbvie, Astellas, Gilead, MSD, Novartis, Pfizer and Sanofi, and has been involved in scientific boards as consultant for Astellas, MSD, and Sanofi. BS received travel grant from Pfizer and MSD, and research grant from bioMérieux. M-EB received travel grants from Pfizer, Astellas and MSD, research grant from Astellas. The remaining authors declare that the research was conducted in the absence of any commercial or financial relationships that could be construed as a potential conflict of interest.
