# Supplementary material for: Clinical Impact of Antifungal Susceptibility, Biofilm Formation and Mannoside Expression of Candida Yeasts on the Outcome of Invasive Candidiasis in ICU: An Ancillary Study on the Prospective AmarCAND2 Cohort
Source: Front Microbiol. 2018 Dec 11;9:2907. doi: 10.3389/fmicb.2018.02907 (PMC6297146; doi:10.3389/fmicb.2018.02907)
Supplement: Supplementary file 1 [file Data_Sheet_1.PDF]

Clinical impact of antifungal susceptibility, biofilm formation and mannoside expression of *Candida* yeasts on the outcome of invasive candidiasis in ICU: an ancillary study on the prospective AmarCAND2 cohort

Gangneux J.-P.[1], Cornet M.[2], Bailly S.[3], Fradin C.[4], Féger C.[5], Timsit J.-F.[3,6], Leroy O.[7], Sendid B.[4,8], and Bougnoux M.-E.[9,10]

Electronic Supplementary Material

Materials and Methods

Yeast identification and distribution per infected sites

Cryopreserved strains were cultured on Chromagar medium (Becton Dickinson, Le Pont-de-Claix, France). After 2 days of growth, they were identified using Matrix Assisted Laser Desorption Ionization - Time of Flight (MALDI-TOF) mass spectrometry (Andromas^{TM}, Paris, France), according to the manufacturer's recommendations as previously described [1].

Antifungal susceptibility testing - Minimum Inhibitory Concentration

For Antifungal susceptibility testing, the cryopreserved isolates were subcultured on Chromagar medium (Becton Dickinson, Le Pont-de-Claix, France) at 35°C for 24h. Then RPMI agar plates were inoculated by a cell suspension adjusted spectrophotometrically to the turbidity of a 0.5 McFarland. Minimum Inhibitory Concentration (MIC) values of the echinocandins (caspofungin (CAS), anidulafungin (ANI), and micafungin (MICA)) and the triazoles (fluconazole (FLZ), voriconazole (VCZ)) were determined. Etests were read after 24 h of incubation at 35°C. The reading end point was 80% of inhibition for triazoles and echinocandins and any growth of microcolonies in a discernable inhibition ellipse was disregarded. Quality control was performed by testing C. krusei ATCC 6258 and C. parapsilosis ATCC 22019 on each day of experiments. Susceptibility categories were determined according to the CLSI Clinical Break Points (CBP) as susceptible (S), intermediate (I) and resistant (R) or the E-coff values (ECV) as wild type (WT) and non-wild type (non-WT) when CBPs are not available [2].

Biofilm formation

Biofilm formation was assessed using the BioFilm Ring Test® (BRT) (BioFilm Control®, Saint-Beauzire, France), by adapting a technique used for bacteria [3]. The adhesion strength of each strain was expressed as BioFilm Index (BFI), according to the dedicated software. The software compares the image of the control well to the image of each well and calculates a corresponding BFI value ranging from 0 to 21. A high BFI value ≥ 15 reflects a complete beads mobility under magnetic action

corresponding to the absence of biofilm formation, while a low value ≤ 3 reflects a complete immobilization of beads embedded in the formed biofilm. According to the BFI, the strains were categorized into three distinct groups: no biofilm producers (BFI≥15), low-biofilm producers (3<BFI <15) and high-biofilm producers (BFI ≤3). Each microbial culture was analyzed in duplicate and experiments were repeated at least two times for each strain to assess repeatability, accuracy, and precision of the assay. Values were considered valid when the standard deviation between duplicates did not exceed 10%. Replicates showed a complete categorical accordance within their classification.

Expression of surface and glycoconjugate mannosides

Yeast growth conditions

Cryopreserved yeast strains were cultured as previously described for 2 days at 30°C on Sabouraud agar plates. The strains were grown for 1 day at 30°C on yeast peptone dextrose plates. The yeast cells were washed twice with cold ultrapure water and aliquoted for ELISA and Western blot assays.

ELISA for the analysis of surface mannoside expression

Yeast cells were suspended in 0.1 M PBS, pH 7.0 containing 4% formalin at a density of 2 x 10^5 cells/ml. Microtiter plates were coated with 100 µl of yeast suspension [4]. After 1 h incubation at room temperature and overnight incubation at 4°C, the wells were washed and blocked with PBS containing 3% bovine serum albumin (BSA; Sigma®, Saint-Quentin Fallavier, France). Surface β-1,2 linked oligomannosides were detected using monoclonal antibody (mAb) 5B2, a rat-mouse IgM specific for β-1,2 linked oligomannosides [5]. The reaction was revealed using horse radish peroxidase (HRP)-labeled anti-mouse IgG. 5B2 and HRP conjugates were diluted 1:5000 and 1:25 000, respectively, in PBS supplemented with 1% BSA, 0.05% Tween 20 (PBST). Peroxidase-labeled concanavalin A (HRP-ConA) diluted 1:2000 in PBST was used to detect α-mannosides. HRP-conjugates were detected with TMB substrate after incubation for 30 min at room temperature in the dark. The reaction was stopped with 1.5 N sulfuric acid, and absorbance was measured at 450 nm. All assays were performed in duplicate. *C. albicans* mutant strain *bmt1*Δ*bmt2*Δ*bmt5*Δ expressing no β -1,2 linked oligomannosides was used as a control [6].

Western blot

Total extracts were obtained from 2 x 10^6 yeast cells, separated by SDS–PAGE [7]and transferred to nitrocellulose membranes as described previously [5, 6]. Membranes were then probed with monoclonal Antibody 5B2 followed by alkaline phosphatase conjugated anti-rat IgM (both diluted 1:2000), or HRP-ConA, as previously described [6].

Results

Yeast species identification

Species and sites of infection are described on **Tables S1 and S2**. The centralized identification using mass spectrometry on strains of cryptic species allowed the identification of 1 strain of *Candida orthopsilosis*, 1 strain of *Candida bracarensis* and 4 strains of *C. dubliniensis*.

Antifungal susceptibility of yeast

The antifungal susceptibility of the 436 strains as determined by the Etest method are presented in **Table S3**. Regarding FLZ, the median MICs obtained were 0.25 µg/ml for *C. albicans*, 4 µg/ml for *C. glabrata*, 0.5 µg/ml for *C. parapsilosis*, 0.75 for *C. tropicalis*; 24 for *C. krusei* and 0.16 for *C. lusitaniae*.

Impact of glycanic derivatives profile

Analysis of the correlation between MS and antifungal susceptibility showed that yeast phenotypes with MS ≤3 were significantly associated with reduced susceptibility to antifungal drugs (I/R profile) ($p<0.001$), while the distribution of strains able or not to produce biofilm is significantly different according to the MS ($p=0.0054$)(**Table S4**).

References

1. Lacroix C, Gicquel A, Sendid B, Meyer J, Accoceberry I, Francois N, Morio F, Desoubeaux G, Chandenier J, Kauffmann-Lacroix C, Hennequin C, Guitard J, Nassif X, Bougnoux ME, (2014) Evaluation of two matrix-assisted laser desorption ionization-time of flight mass spectrometry (MALDI-TOF MS) systems for the identification of Candida species. Clinical microbiology and infection : the official publication of the European Society of Clinical Microbiology and Infectious Diseases 20: 153-158
2. CLSI (2012) Clinical and Laboratory Standards Institute (CLSI) Reference method for broth dilution antifungal susceptibility testing of yeasts; fourth informational supplement. In: Editor (ed)^(eds) Book Clinical and Laboratory Standards Institute (CLSI) Reference method for broth dilution antifungal susceptibility testing of yeasts; fourth informational supplement. Clinical and Laboratory Standards Institute, City, pp.
3. Olivares E, Badel-Berchoux S, Provot C, Jaulhac B, Prevost G, Bernardi T, Jehl F, (2016) The BioFilm Ring Test: a Rapid Method for Routine Analysis of Pseudomonas aeruginosa Biofilm Formation Kinetics. Journal of clinical microbiology 54: 657-661
4. Fruit J, Cailliez JC, Odds FC, Poulain D, (1990) Expression of an epitope by surface glycoproteins of Candida albicans. Variability among species, strains and yeast cells of the genus Candida. Journal of medical and veterinary mycology : bi-monthly publication of the International Society for Human and Animal Mycology 28: 241-252

5. Trinel PA, Faille C, Jacquinot PM, Cailliez JC, Poulain D, (1992) Mapping of Candida albicans oligomannosidic epitopes by using monoclonal antibodies. Infection and immunity 60: 3845-3851
6. Courjol F, Jouault T, Mille C, Hall R, Maes E, Sendid B, Mallet JM, Guerardel Y, Gow NA, Poulain D, Fradin C, (2015) beta-1,2-Mannosyltransferases 1 and 3 Participate in Yeast and Hyphae O- and N-Linked Mannosylation and Alter Candida albicans Fitness During Infection. Open forum infectious diseases 2: ofv116
7. Laemmli UK, (1970) Cleavage of structural proteins during the assembly of the head of bacteriophage T4. Nature 227: 680-685

Table S1 – description of the species of *Candida* isolated from the 349 patients of the AmarCAND2

Candida species	Number (%)
C. albicans	285 (65.4)
C. glabrata	65 (14.9)
C. parapsilosis	20 (4.6)
C. tropicalis	20 (4.6)
C. krusei	11 (2.5)
C. lusitaniae	10 (2.3)
C. guillermondii	4 (0.9)
C. dubliniensis	3 (0.7)
Other	18 (4.1)
Total	436 (100%)

Other = *C. norvegiensis; C. famata; C. inconspicua; C. pulcherrina; C. utilis*

Table S2 - Yeast species and site of infection

	Peritoneal fluid	Blood culture	Other sample*	Total (%)
C. albicans	137 (67.1)	108 (64.7)	40 (61.5)	285 (65.4)
C. glabrata	31 (15.2)	25 (15)	11 (16.9)	65 (14.9)
C. parapsilosis	2 (1.0)	17 (10.1)	2 (3.1)	20 (4.6)
C. tropicalis	12 (5.6)	3 (1.9)	6 (9.2)	20 (4.6)
C. krusei	9 (4.6)	1 (0.6)	1 (1.5)	11 (2.5)
C. lusitaniae	5 (2.5)	5 (2.9)	-	10 (2.3)
C. guillermondii	-	3 (1.8)	1 (1.5)	4 (0.9)
C. dubliniensis	-	2 (1.1)	1 (1.5)	3 (0.7)
Other species**	8 (4.0)	3 (1.9)	3 (4.6)	18 (4.1)
Total	204 (46.8)	167 (38.3)	65 (14.9)	436 (100%)

*Other samples : biopsies and pleural fluid

**Other species = *C. norvegiensis; C. famata; C. inconspicua; C. pulcherrina; C. utilis; C. orthopsilosis; C. bracarensis*

Table S3 - MIC values (µg/ml) determined by Etest method for triazole and echinocandin antifungal agents of the main *Candida* species strains of the AmarCAND2 study

Species	Fluconazole			Voriconazole			Anidulafungin			Micafungin			Caspofungin		
(No. of strains)	Median [IQR]	MIC50	MIC90	Median [IQR]	MIC50	MIC90	Median [IQR]	MIC50	MIC90	Median [IQR]	MIC50	MIC90	Median [IQR]	MIC50	MIC90
C. albicans (285)	0.25 [0.19; 0.5]	0.25	1	0.008 [0.006; 0.016]	0.008	0.047	0.003 [0.003; 0.006]	0.003	0.008	0.016 [0.012; 0.023]	0.016	0.032	0.064 [0.032; 0.094]	0.064	0.19
C. glabrata (65)	4 [2 ; 8]	4	12	0.094 [0.047; 0.125]	0.094	0.25	0.012 [0.008; 0.016]	0.012	0.016	0.016 [0.012; 0.023]	0.016	0.032	0.19 [0.094; 0.19]	0.19	0.25
C. parapsilosis (20)	0.5 [0.38; 1.75]	0.5	28	0.012 [0.007; 0.032]	0.012	0.079	0.38 [0.020; 0.625]	0.38	1.75	0.5 [0.19; 0.875]	0.5	1.25	0.44 [0.19; 0.75]	0.44	1
C. tropicalis (20)	0.75 [0.44; 1.25]	0.75	7	0.032 [0.016; 0.064]	0.032	0.094	0.012 [0.008; 0.012]	0.012	0.032	0.032 [0.028; 0.032]	0.032	0.0945	0.125 [0.094; 0.25]	0.125	0.25
C. krusei (11)	24 [16; 48]	24	48		0.125	0.25	0.023 [0.016; 0.023]	0.023	0.032	0.19 [0.125; 0.19]	0.19	0.19	0.38 [0.25; 0.5]	0.38	0.5
C. lusitaniae (10)	0.1575 [0.047; 0.25]	0.16	0.625	0.004 [0.004; 0.006]	0.004	0.012	0.012 [0.008; 0.016]	0.012	0.0275	0.064 [0.032; 0.064]	0.064	0.125	0.094 [0.064; 0.25]	0.094	0.25
Other *Candida* species** (25)	0.24 [0.094; 4]	0.24	16	0.012 [0.004; 0.064]	0.012	0.125	0.008 [0.008; 0.023]	0.008	0.25	0.064 [0.032; 0.094]	0.064	0.19	0.125 [0.064; 0.19]	0.125	0.38

* IQR= interquartile range ** Other = *C. guillermondii*; *C. dubliniensis; C. norvegiensis; C. famata; C. inconspicua; C. pulcherrina; C. utilis*

Table S4 - MS values for the different *Candida* species

Candida species	No. of isolates	% of isolates	MS values				
			Mean	SD	Q1	Median	Q3
C. albicans	259	66.9	17.9	31.3	3.20	7.7	21.8
C. glabrata	56	14.5	2.5	8.4	0.4	0.8	1.9
C. parapsilosis	19	4.9	1.5	6.3	0.0	0.0	0.1
C. tropicalis	16	4.1	38.6	36.7	4.3	30.7	55.3
C. krusei	12	3.1	0.1	0.1	0.0	0.1	0.1
C. lusitaniae	8	2.1	142.4	100.5	55.0	142.4	222.6
C. dubliniensis	4	1.0	16.1	1.2	15.4	15.8	16.7
C. guillermondii	3	0.8	1.1	0.7	0.7	0.8	1.9
Miscellaneous	10	2.6	10.4	32.0	0.0	0.0	0.3

MS: mannoside score; SD: standard deviation; Q1, Q3: 1st and 3rd quartiles
